# Optimization of Reconfigurable Satellite Constellations Using Simulated Annealing and Genetic Algorithm

**DOI:** 10.3390/s19040765

**Published:** 2019-02-13

**Authors:** Sung Wook Paek, Sangtae Kim, Olivier de Weck

**Affiliations:** 1 Materials R&D Center, Samsung SDI, Gyeonggi-do 16678, Korea; 2 Center for Electronic Materials, Korea Institute of Science and Technology, Seoul 02792, Korea; stkim@kist.re.kr; 3 Department of Aeronautics and Astronautics, Massachusetts Institute of Technology, Cambridge, MA 02139, USA; deweck@mit.edu

**Keywords:** Earth observation, remote sensing, satellite constellation, reconfigurability, repeat ground tracks, simulated annealing, genetic algorithm

## Abstract

Agile Earth observation can be achieved with responsiveness in satellite launches, sensor pointing, or orbit reconfiguration. This study presents a framework for designing reconfigurable satellite constellations capable of both regular Earth observation and disaster monitoring. These observation modes are termed global observation mode and regional observation mode, constituting a reconfigurable satellite constellation (ReCon). Systems engineering approaches are employed to formulate this multidisciplinary problem of co-optimizing satellite design and orbits. Two heuristic methods, simulated annealing (SA) and genetic algorithm (GA), are widely used for discrete combinatorial problems and therefore used in this study to benchmark against a gradient-based method. Point-based SA performed similar or slightly better than the gradient-based method, whereas population-based GA outperformed the other two. The resultant ReCon satellite design is physically feasible and offers performance-to-cost(mass) superior to static constellations. Ongoing research on observation scheduling and constellation management will extend the ReCon applications to radar imaging and radio occultation beyond visible wavelengths and nearby spectrums.

## 1. Introduction

Earth observation has experienced unprecedented growth through the use of satellite data [1,2]. Space-based, spatio-temporal data is now regularly used to remotely measure fresh water elevation [3], explore potential mineral deposits [4], monitor changes in land-cover and land-use [5,6], to name a few practical applications. In particular, Earth observation for situational awareness often involves mobile targets, such as hurricanes [7,8] or emergency areas whose locations cannot be determined a priori [9,10]. For example, crop classification and growth monitoring may be routinely performed on pre-designated areas [11,12], whereas the time and location of flooding or drought cannot be accurately predicted and requires contingent responses. 

**Figure 1:**
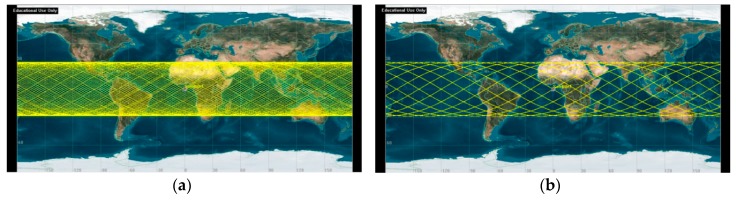
Two observation modes of a ReCon: (**a**) Global observation mode (GOM); (**b**) Regional observation mode (ROM). Note that the satellite is moving along solid lines from left to right in prograde orbits (this case) and vice versa in retrograde orbits.

As a way of incorporating responsiveness and agility into Earth observation, the concept of reconfigurable satellite constellation (ReCon) has been proposed [13,14]. The operation of a ReCon comprises the following two modes: Global observation mode (GOM) for normal operations and regional observation mode (ROM) for contingent responses. In GOM, satellites in a ReCon evenly scan the entire region within a latitude band without any bias (Figure 1a). In ROM, satellites move to repeat ground tracks (RGT) and periodically fly more often over certain regions along their fixed paths (Figure 1b) while ignoring the remainder in between. The satellites in ROM have 2.5 times more access time than those in GOM on average [15]. Near the equator where the satellites struggle in terms of GOM access, ROM achieves up to 10 times more access, which is achieved at the intersections of ascending trajectories and descending trajectories depicted in Figure 1b. There are a few examples that may be regarded as precursors of a ReCon. Two European Remote Sensing satellites, ERS-1 and ERS-2, were lunched into a single orbit and could reconfigure the revisit time of their repeat ground tracks from 3 to 35 days [16,17]. The KH(keyhole)-11 satellites consisted of five satellites allocated to two planes, all of which were highly maneuverable for reconnaissance purposes [18]. Further scale-up of a ReCon would require optimal planning of multiple satellite maneuvers. The ReCon just started gaining interests from industries, out of theory, as its operational complexity is being overcome via onboard algorithms and ground computing resources [19,20,21]. Satellite orbits and view orientation can thus be planned in time to maximize the value of distributed satellite missions (DSMs) [22,23,24,25].

Walker Delta and Walker Star are well-known constellation types that symmetrically distribute satellites in the inertial reference frame to provide global coverage [26,27]. The former distributes orbit planes over the full longitudes of 360 degrees while the latter distributes over the longitude range of 180 degrees because of near-polar orbital inclination. Flower Constellations (FCs) distributes satellites in a rotating reference frame instead of an inertial reference frame [28]. Sub-global coverage may also be provided by squeezing orbital plane distribution onto a confined longitude band of interest [5,29]. The above-mentioned methodology has been implemented in a number of DSM tools. For example, Operational Network of Individual Observation Nodes (ONION) aims at simultaneously meeting Earth observation requirements and data download requirements, whose optimal solution is a hybrid Walker constellation of small satellites (CubeSats) and larger satellites [30]. Trade-space Analysis Tool for Constellations (TAT-C) conducts performance-cost analyses on uniform Walker constellations, non-uniform Walker constellations, and ad-hoc constellations [31]. The proposed ReCon framework may supplement these tools by adding reconfigurability to static Earth observing constellations operating in various wavelengths.

This study focuses on heuristic optimization techniques, simulated annealing and genetic algorithm which are representative of single-point-based methods and population-based methods, respectively. The two methods have widely been used in solving NP-hard combinatorial problems [32,33] to benchmark each other, sometimes forming hybrids to complement one another [34,35]. These two are a good starting point before transitioning to multiple-objective optimization or other state-of-the-art methods in future work [36,37].

The paper is organized as follows. Section 2 delineates the ReCon framework, and Section 3 conducts a preliminary design search prior to applying optimization methods. After that, Section 4 and Section 5 apply optimization techniques of simulated annealing and genetic algorithm, respectively. Section 6 analyzes and compares the results from the previous three sections, and conclusions are made in Section 7.

## 2. Methodology

The proposed ReCon framework employs multidisciplinary system design optimization (MSDO) derived from systems engineering. The objective of optimizing a ReCon is threefold: To minimize revisit access time and maximize area coverage; to minimize the initial launch mass of the entire satellite constellation (number of orbit planes × number of satellites per plane × satellite mass); and to minimize reconfiguration time. The ReCon framework optimizes the geometry of an individual orbit and the arrangement of multiple orbits in addition to satellite payload design while satisfying the imagery resolution requirement.

### 2.1. Problem Formulation

The ReCon framework may be regarded as nonlinear programming (NLP), due to the nonlinear nature of satellite coverage and launch mass. Equation (1) is a general NLP formulation where the fitness function *F* or the constraint ***h*** is nonlinear. Both the constraint and the objective are nonlinear in the ReCon framework, as will be discussed in more detail in Section 2.2.4 and Section 2.2.5. Multiple constraints are expressed as a vector ***h***, and the objective *F* is scalar. The design vector ***x*** (Section 2.2.1) is varied throughout the optimization process such that ***x***_LB_ ≤ ***x*** ≤ ***x***_UB_, but the operating parameter ***p*** remains unchanged (Section 2.2.3). Intermediate variables (Section 2.2.2) are not included in Equation (1), but they play a role as data inputs and output to relay internal states among subroutines.

(1) MinJ(x,p) s.t. h(x,p)≤0  ,

Inequality constraint may be enforced by adding penalization to J where that larger values for penalty vector elements will more strongly discourage constraint violation. In Equation (2), each constraint violation is weight-accumulated only if *h_i_* is positive as shown by indicator function **1**_h>0_.

(2) $$\mathrm{Min}J\left(x,p\right)+g\sum_{i}\{w_{h_{i}}h_{i}\left(x,p\right)1_{h_{i}>0}\}\;,$$

Finally, non-penalized *J* is also a weighted sum of several figures of merit, as shown in Equation (3). Definitions for constraints and figures of merit are summarized in Table 1.

(3) $$\mathrm{Min}\sum_{i}\{w_{F_{i}}F_{i}\left(x,p\right)\}+g\sum_{i}\{w_{h_{i}}h_{i}\left(x,p\right)1_{h_{i}>0}\}\;.$$

It would be noteworthy to mention that these figures of merit exhibit highly nonlinear behaviors and lack closed forms most of the time. Among four figures of merit, only reconfiguration time, as will be discussed in Section 2.3.3, has a closed form solution which is inversely proportional to the product of (Earth radius + altitude) and ∆(altitude). Constellation mass is obtained through numerical iterations described in Section 2.3.4 to solve the rocket equation with exponential terms. The remaining two figures of merit are temporal coverage in GOM and revisit time in ROM. A potential region of interest (latitude band in this case) should be sufficiently observed by maximizing the temporal coverage in GOM, and an outstanding target within that region, once identified, must be visited as frequently as possible by minimizing the revisit time in ROM. There have been efforts to develop non-numerical algorithms for calculating the temporal coverage and revisit time of ground targets at the equator [38], but considerable errors would occur if targets are located at high latitudes where orbit groundtracks are cluttered (Figure 1). These methods also apply only to a single satellite, yet to be extended up to a constellation level. Numerical orbit simulation is therefore used in this work to compute coverage and revisit time. While coverage and revisit time are enhanced as the number of satellites increases, the larger launch mass (proxy of cost) must be accompanied. Combined with the inherent nonlinearity of each figure of merit, tensions among figures of merit lead to complex objective space of this problem, discussed again in Section 6.1 and exemplified by the poor performance of gradient-based approaches. 

Constraints are intended to bring engineering considerations into the optimization process such that the final solution is physically reasonable, as well as being mathematically optimal. Minimum and maximum altitude boundaries define design space where Earth-observing satellite can safely operate without suffering excessive atmospheric drag or radiation. Maximum aperture and propellant fraction limits are prescribed to ensure that ReCon satellites can be readily manufactured with current technologies and be safely launched in reality. 

### 2.2. Model Overview

Concurrent design of satellite size and orbit configuration needs several design variables; without enough number of them, many aspects of the tradespace cannot be considered. On the other hand, considering too many variables would increase the tradespace beyond our computing power and slow down the optimization process. To achieve a balance between a coarse model and a fine model in fast prototyping, this study uses the following five data types: Design variables, parameters, internal variables, constraints, and objectives which will be explained in this subsection.

#### 2.2.1. Design Variables

Design variables are analogous to knobs used for adjusting design specifications. The following five design variables have been chosen to maintain adequate degrees of freedom:

- Altitude;
- Altitude difference;
- Number of orbit planes;
- Number of satellites per plane;
- Field of regard.

The altitude variable is the height of a satellite in ROM represented by a repeat ground track (RGT) ratio. For example, a ratio of 15/1 means that satellite revolves around Earth 15 times in 1 day (Earth rotates once). As seen in Table 2, ratios between 31/2 and 14/1 are sampled such that the resulting ROM has an altitude between 300 km and 1200km. The procedures for calculating RGT altitudes are provided in Appendix A [39,40]. The altitude difference is the difference between the GOM altitude the ROM altitude, i.e., (ROM altitude) + (altitude difference) = (GOM altitude). While these first two variables govern the vertical distribution of satellites, the next two variables dictate the horizontal distribution of satellites. Lastly, the field of regard relates to the viewing range, achieved by either maneuvering the whole satellite or the optics subsystem only.

#### 2.2.2. Internal Variables

Internal variables are intermediate values generated through arithmetic operations on design variables. They are still variables, whose values are affected by design inputs, but cannot be directly manipulated by a user, as the term “internal” implies. For example, the user cannot directly control the dimensions of remote sensing optics, but the combination of satellite altitude, field of regard, and GSD requirement determines the necessary dimensions of optical instruments. The list of internal variables is provided in Table 3.

#### 2.2.3. Parameters

Parameters are non-varying constants whose values are fixed for simplicity or quick comparison of design architectures. For example, a total of 10 reconfiguration moves from GOM to ROM or vice versa are permitted over a 5-year lifetime, as shown in Table 4. The ground resolution of 0.5 m is also assumed which must be satisfied even when a satellite is tilted the farthest and its distance to the ground is the largest. The Walker phasing parameter of 1 means that the satellites in adjacent orbital planes are separated by 360/*N_s_* × 1 degrees where *Ns* is the total number of satellites in a constellation [41]. The regional latitude of interest is set to be the 55^th^ parallel of North, assuming a wildfire monitoring mission over Canada and Alaska [42,43].

#### 2.2.4. Constraints

Constraints are maximal or minimal boundaries desired for intermediate variables. Since the user has no direct control over them, the values of intermediate variables are indirectly regulated by penalization if their values exceed or fall below constraints, as summarized in Table 5. The field of regard, defined as the maximum half-cone tilt angle from nadir, is usually 30° and 45° in some cases, making 50° an appropriate constraint [44,45]. The minimum altitude is set as 350 km below which atmospheric friction becomes prohibitive, and the maximum altitude is set as 1200 km beyond which ground resolution requirements are harder to achieve.

#### 2.2.5. Objectives

The goals of a ReCon are to sufficiently observe a potential region of interest (maximize temporal coverage) and to visit a selected target as often as possible (minimize revisit time) at the same time. Revisit time and temporal coverage in ROM and GOM each yield the four possible objectives related to observation performance, but only ROM revisit time and GOM coverage are considered, as shown in Table 6. Reconfiguration time should also be minimized for the agile transition between GOM and ROM, taking preferably several days rather than weeks. Constellation mass is directly related to the cost of developing, manufacturing, and launching remote sensing satellites. Although the detailed cost analysis is not the scope of this study, providing better observation performance while suppressing the satellite mass (and cost) is considered ideal.

### 2.3. Simulation Model

**Figure 2:**
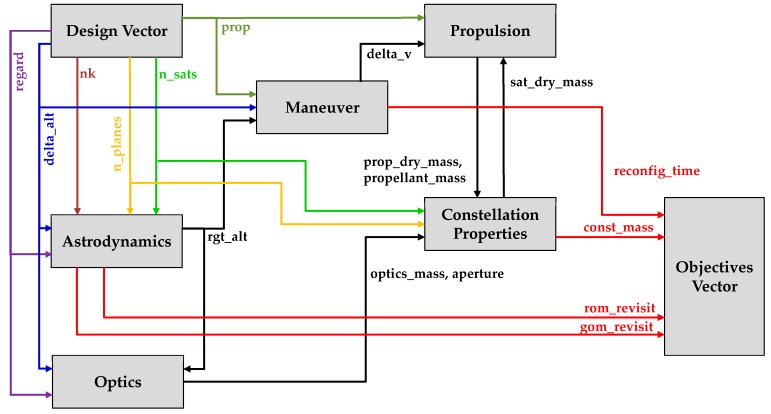
Data flow in ReCon simulation.

The data types discussed so far are input to, exchanged between, or output from modules consisting the entire simulation. Each module corresponds to one subsystem of a remote sensing satellite. In general, the following subsystems are of special interest: Optics subsystem for Earth observation; propulsion subsystem for stationkeeping; and guidance, navigation, and control (GNC) subsystem for determination of satellite orientation and location. These subsystems correspond to optics, propulsion, and astrodynamics modules in Figure 2, whose inputs and outputs are summarized in Table 7.

#### 2.3.1. Astrodynamics Module

The astrodynamics module initializes basic orbit parameters first and calculates satellite trajectories over time. The trajectory of an Earth-orbiting satellite is described using six parameters or orbital elements: *a*, *e*, *i*, *Ω*, *ω*, and *ν* [1]. The first two elements define the geometry of an orbit, semi-major axis (*a*) for size and eccentricity (*e*) for shape. The next two elements define the three-dimensional orientation of an orbit. Inclination (*i*) defines the tilting angle of a satellite’s orbit plane with respect to Earth’s equator, and the longitude of the ascending node (*Ω*) defines the direction of tilting (location of ascending nodes) relative to the vernal equinox position. For an Earth-centered orbit, the longitude of the ascending node is also referred to as the right ascension of the ascending node (RAAN). From the ascending node, the argument of perigee (ω) is measured, from which the satellite’s position is determined with the true anomaly (ν).

**Figure 3:**
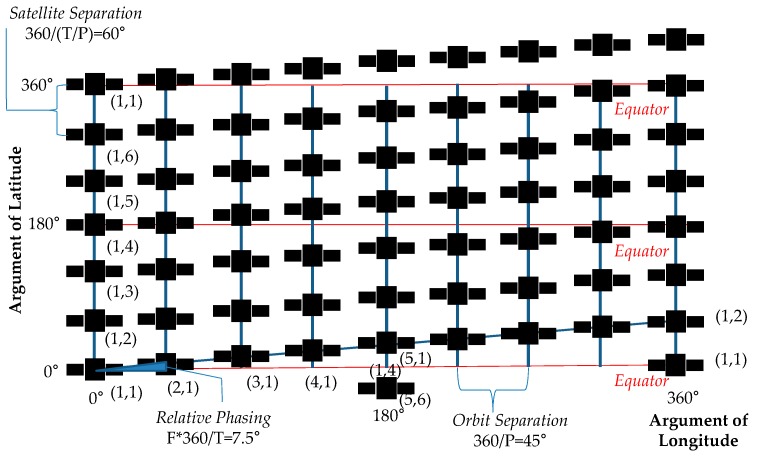
Walker pattern of Globalstar satellite constellation.

One of the popular ways to design circular orbit constellations is the Walker Delta constellation (termed Walker constellation hereafter), which provides the most symmetry by having similar orbits amongst satellites. This maximal symmetry not only greatly reduces design space, but also provides advantages in constellation management because any satellite in a constellation undergoes similar effects from orbit decaying or other perturbations. The Walker constellation uses a notation of *i:T/P/F* where a total of T satellites are evenly distributed in *P* orbit planes inclined at *i* degrees and separated from one another by 360/*T* × *F* degrees. Figure 3 shows a Globalstar constellation which uses a 48/8/1 Walker pattern. The phasing parameter *F* can take integer values from 0 to *P*−1 [46].

**Figure 4:**
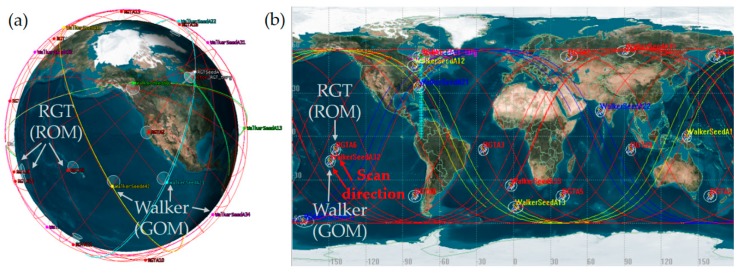
ReCon simulations: (**a**) 3D view; (**b**) 2D view.

A three-dimensional Earth-centered view is shown in Figure 4a where ROM orbits and GOM orbits are represented by thin red lines and thick lines, respectively. Figure 4b depicts ground tracks instead, clearly showing deviation between ROM groundtracks and GOM groundtracks, due to altitude difference. Because there is no specific target of interest in GOM, the coverage statistics were gathered over the entire latitude band in the northern hemisphere which can be reached by the satellites, between the equator and 60° N, which is marked by light blue grids in the figure. A target of interest during ROM has the latitude of 55° N as mentioned earlier.

The RGT orbits considered here repeats after two days at most, so the STK simulations were run for two days in simulation time. The trajectory calculation considers up to J4 effects in this study.

#### 2.3.2. Optics Module

**Figure 5:**
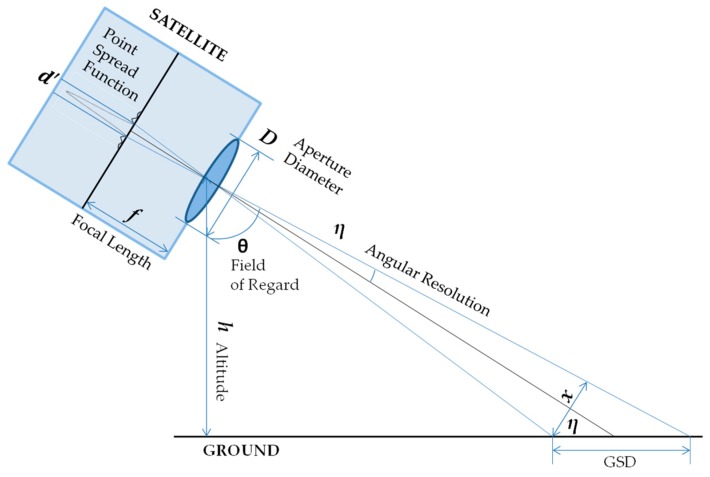
Optical relationship of ground sample distance (GSD) with other parameters (not to scale).

In remote sensing, ground sample distance (GSD) is used as a metric of image resolution and refers to the distance between the centers of digital photo pixels projected on the ground. Other terms, such as ground-projected sample interval (GSI) and ground-projected instantaneous field of view (GIFOV) may be used interchangeably [47]. This separation requirement for resolving two ground points can be translated into the satellite aperture diameter if the optics system is limited only by diffraction, not by the lens imperfections or the pixel size [48]. Diffraction limit is illustrated as *d*’ in Figure 5 where the standard Rayleigh diffraction criterion limits angular resolution to:

(4) $$\theta =1.22\frac{\lambda }{D}\;\;,$$

The angular resolution (*θ*) is proportional to the wavelength (λ) and inversely proportional to the aperture diameter (*D*). The same angular resolution may also be expressed in term of the radius of the first Airy ring, i.e., the distance between the first lobes from the center of point spread function:

(5) $$\theta =\frac{d^{\prime }}{2f}=\frac{x}{2R_{s}}\;\;,$$

where *f* is the focal length, *h* is the satellite altitude, and *x* is the ground sample distance (GSD) when the satellite is pointing perpendicular to the ground (nadir direction).

When the observation payload is oriented at the edge of its field of regard, tilted with an off-nadir angle *η*, the satellite-to-ground distance increases to *R*_s_ = *h*/cos(*η*). Retaining the same resolution therefore requires a larger aperture than the nadir case, which can be calculated by replacing *h* with *R*_s_ in Equation (6) [49]:

(6) $$D=2.44\frac{\lambda R_{s}}{x}=2.44\frac{\lambda h}{xcos\eta }\;\;,$$

The ground projection of a pixel (*x*) is also elongated into GSD per relationship *x* = GSDcos(*η*):

(7) $$D=2.44\frac{\lambda h}{GSDcos^{2}\eta }\;\;,$$

where GSD = 1 m and *λ* = 500 nm. This aperture diameter is used to calculate the mass of the sensor payload, including the primary mirror, optical telescope assembly (OTA), imagers, and supporting mechanical and electronic components. The mass calculation is done by considering an empirical relationship between the payload mass and the aperture size, as shown in Equation (8) where the mass is in kilograms and the aperture diameter is in meters. Similarly, Equation (9) describes the relationship between the aperture size and the non-sensor platform mass of a satellite. Unlike *m_dry,optics_* which increases super-linearly (exponent greater than 1) with aperture in Equation (7), *m_dry,non-optics_* increases almost linearly (exponent close to 1) with aperture. The data used to derive the power law relationship is provided in Appendix B [50].

(8) $$m_{sensor\_payload}=m_{dry,optic}=418D^{1.37}\left[\mathrm{kg}\right]\;\;,$$

(9) $$m_{platform}=m_{dry,non-optic}=754D^{1.03}\left[\mathrm{kg}\right]\;\;,$$

#### 2.3.3. Maneuvers Module

**Figure 6:**
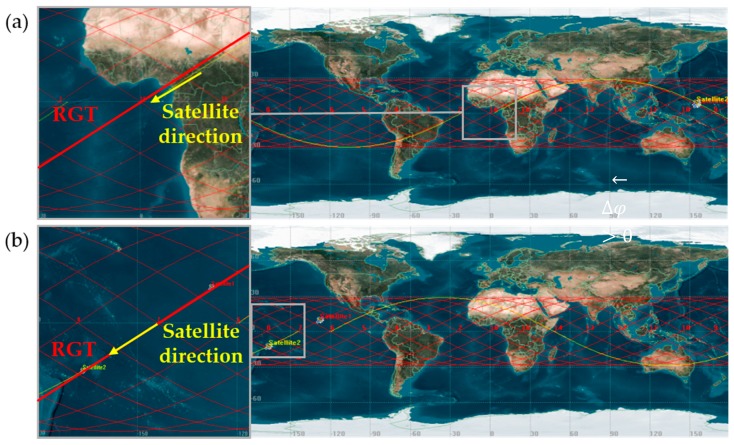
Reconfiguration time: (**a**) Target identification at t = T_0_ (satellite not aligned with repeat groundtracks); (**b**) Orbit transfer from GOM to ROM at t = T_0_ + T_R_ (satellite aligned with repeat groundtracks).

The maneuvers module calculates reconfiguration time which is one of the multiple objectives in ReCon optimization. It is defined as a time elapse from the identification of a target (Figure 6a) until the alignment of GOM and ROM groundtracks (Figure 6b). Once aligned, the satellite is ready for switching observation modes; the time required for trajectory switching through a Hohmann transfer (less than an hour) is negligible compared to the waiting time for groundtracks to be naturally aligned (several days). 

Given a ROM altitude of *h* and a GOM altitude of *h* + ∆*h*, their orbital periods differ by:

(10) $$\Delta T=2\pi \left(\sqrt{\frac{{\left(R_{E}+h+\Delta h\right)}^{3}}{\mu_{E}}}-\sqrt{\frac{{\left(R_{E}+h\right)}^{3}}{\mu_{E}}}\right)\;\;,$$

If ∆*h* > 0, a satellite in GOM will take a longer time to complete one revolution than a satellite in ROM. This lagging causes a westward drift of the NRGTs relative to the ground-fixed RGTs. The distance by which NRGTs deviate from RGTs at equator after one orbit is [13]:

(11) $$\Delta d=\left(\omega_{E}-\dot{\Omega }\right)R_{E}\Delta T\;,$$

If a satellite in ROM orbits the Earth *N_p_* times in *N_d_* days (RGT ratio *N_p_*/*N_d_*), the deviation distance along the equator in is *N_p_*∆*d* in *N_d_* days, or *N_p_*∆*d*/*N_d_* per day. Finally, the reconfiguration time measured in days for separation distance *d* or separation angle *φ* is:

(12) $$T_{R}=\frac{N_{d}d}{N_{s}\Delta d}=\frac{N_{d}R_{E}\Delta \varphi }{N_{s}\left(\omega_{E}-\dot{\Omega }\right)R_{E}\Delta T}\left[\mathrm{day}\right]\;,$$

Finally, ∆*h* is also used in this module to calculate delta-v (metric of fuel consumption). In Equation (12), the reconfiguration term grows if ∆*h* is increased. The expressions for the reconfiguration term and others are provided in Appendix C [51].

(13) $$\Delta V=\Delta V_{commisioning}+\Delta V_{reconfig}+\Delta V_{stationkeeping}+\Delta V_{decommisioning}\left[\mathrm{day}\right]\;.$$

#### 2.3.4. Propulsion and Constellation Modules

**Figure 7:**
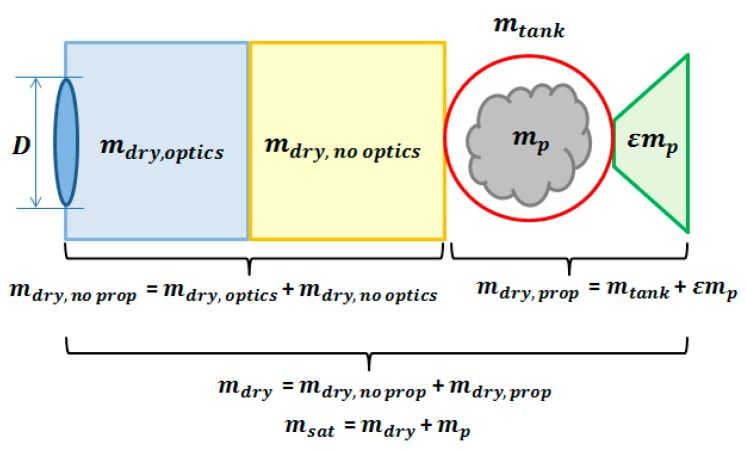
Mass breakdown of a ReCon satellite.

Because a ReCon is intended for highly agile Earth observation, its satellite mass is largely broken down into the propulsive part and the non-propulsive part. First, the propulsive mass comprises the propellant mass (*propellant_mass*) and the propulsion subsystem mass (*prop_dry_mass*), which are calculated by the Propulsion Module in Table 2, Figure 2, and Table 6. The propulsion subsystem mass is further broken down into the tankage mass (*m_tank_*) and the valve and nozzle mass (ε*m_p_*) which is proportional to the propellant mass (*m_p_*) as depicted in Figure 7. Second, the non-propulsive mass (*m_dry,non-optics_*) is calculated by the Constellation Module. It consists of the optics subsystem and the non-optics subsystem; the term “non-optics” here collectively refer to parts for communications, electric power management, etc.

The empirical relationship between *m_tank_* and *m_p_* are given in Equations (14) and (15) for two propellant types, cold gas and monopropellant respectively. The dataset and fitting lines are provided in Appendix B. It is also notable that the exponent value of 0.59 is close to 0.67 = 2/3 indicating that the mass of propellant tank, basically a thin shell, grows a two-dimensionally while its volume grows three-dimensionally.

(14) $$m_{tank\left(cold\right)}=61.7V_{tank}{}^{0.594}=\;61.7{\left(\frac{m_{p}}{\rho_{p}}\right)}^{0.594}\left[\mathrm{kg}\right],$$

(15) $$m_{tank\left(mono\right)}=38.7V_{tank}{}^{0.592}=\;38.7{\left(\frac{m_{p}}{\rho_{p}}\right)}^{0.592}\left[\mathrm{kg}\right],$$

With the aforementioned terms, the satellite mass can be calculated using the Tsiolkovsky rocket equation, as shown in Equation (16). Solving the equation requires numerical iterations, such as the Newton-Rapshon method, indicative of tight coupling between the Propulsion Module and Constellation Module.

(16) $$\frac{m_{dry,optics\;}+\;m_{dry,non-optics}\;+\;m_{tank}\;+\;\left(\varepsilon \;+\;1\right)m_{p}}{m_{dry,optics\;}+\;m_{dry,non-optics}\;+\;m_{tank}\;+\;\varepsilon m_{p}}=e^{g\Delta V/I_{sp}}\;\;,$$

## 3. Preliminary Sampling and Analysis

**Figure 8:**
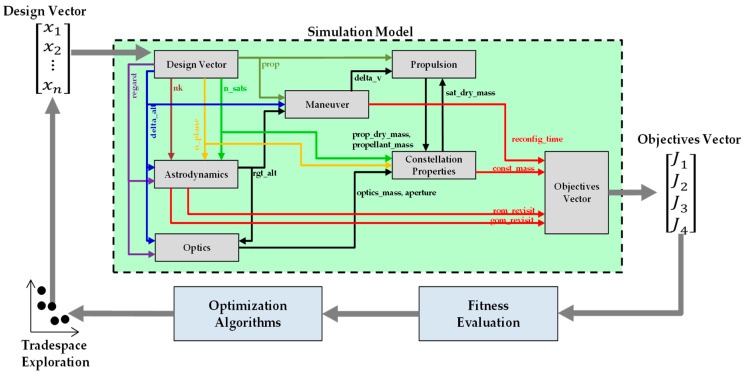
ReCon optimization framework.

With aforementioned subsystem modules, the ReCon simulation for agile Earth observation may now be used in combination with optimization algorithms, as depicted in Figure 8. Optimization algorithms are located at a feedback loop to evaluate the current objectives vector and to decide the next design vector.

**Figure 9:**
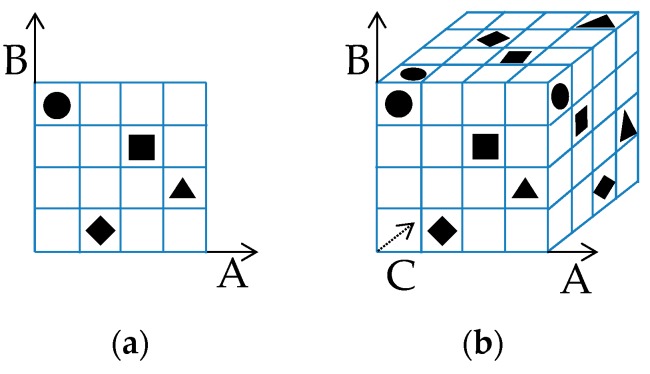
Latin hypercubes: (**a**) Two factors (A, B) and four levels; (**b**) three factors (A, B, C) and four levels.

Latin hypercube sampling (LHS) is a technique that can reduce the number of sampling while maintaining overall coverage. LHS divides the design space into *l* divisions (levels of value) for each of *n* factors (variables), combining them randomly. A square grid containing samples is called a Latin square if and only if there is only one sample in each row and each column (Figure 9).

In ReCon, five factors were used because the sixth variable, propellant type, had been fixed as a monopropellant. Table 8 shows the factors and levels used for the experiment: Four levels for *n*/*k* ratio (*N*), number of planes (*P*), and number of satellites per plane (*S*); eight levels for the Walker altitude difference (*A*) and FoR (*R*). 

A Latin hypercube sample of 100 design points was created using a MATLAB built-in function, *lhsdesign*. At each point, the following four figures of merit are compared to their corresponding 100-point average: ROM revisit time, GOM revisit time, constellation mass, and reconfiguration time. LHS reduced the total of 4096 combinations down to 100 design points via LHS to evaluate the following figures of merit: ROM revisit time, GOM revisit time, constellation mass, and reconfiguration time. Table 9 shows the main effects of the levels of each factor, where blue boxes and red boxes indicate the level of a given factor that has the greatest effect in a positive direction and in a negative direction, respectively. Note that all metrics are better if their values are smaller. The following trends can be observed from the LHS results:

- As the RGT ratio decreases, the mass of the entire constellation increases because more propellant is required (i) to raise the altitude of satellites from the parking orbit to higher altitudes at the beginning of life and (ii) to lower the altitude to the disposal orbit. Reconfiguration time also increases with the RGT ratio because there are fewer locations where reconfiguration can occur.
- High altitude difference increases both ROM revisit time and GOM revisit time because a satellite has to orbit along a longer trajectory with a lower orbit velocity, which leads to a longer orbit period. The constellation mass decreases as altitude difference increases, mainly, due to lower atmospheric drag and subsequent reduction in propellant mass. The reconfiguration time decreases as the absolute value of altitude difference increases because a greater deviation from the Walker altitude makes the orbit plane drift faster.
- Both ROM revisit time and GOM revisit time (to a lesser extent) decrease when the number of planes decreases and the number of satellites per plane increases.
- Increasing the FoR decreases the constellation mass.

From which the following is recommended regarding a starting point for optimization algorithms:

- The number of revolutions per day should be large.
- The altitude difference from the Walker constellation should be large.
- The satellites should be distributed in a small number of orbit planes.
- The FoR should be large.

By satisfying these initial conditions, the starting point could be located as close to optima as possible to save computation time and improve the quality of solutions.

## 4. Simulated Annealing

**Figure 10:**
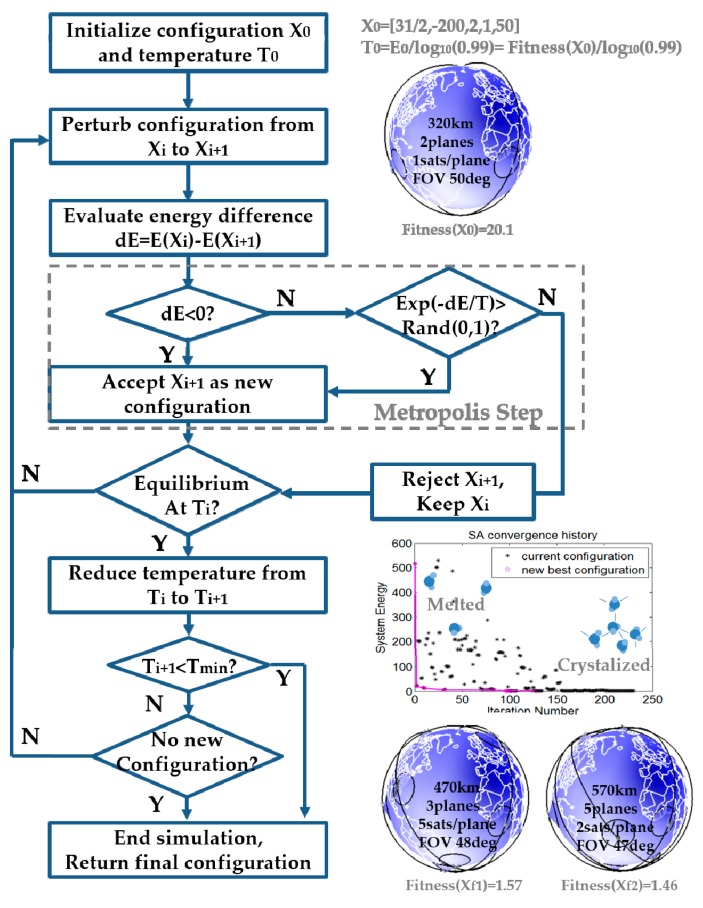
Simulated Annealing (SA) algorithm.

Simulated Annealing (SA) is an optimization algorithm named and inspired by an annealing treatment in metallurgy. Originally, annealing is a cooling technique that increases the crystal sizes and reduces their defects by letting atoms settle down to a minimum energy state. SA attempts to computationally mimic this physical phenomenon through perturbing the existing configuration and accepting the new configuration with a probability dependent upon both energy difference and the system temperature. It is the Metropolis-Hastings algorithm that determines whether or not to accept a new configuration. A lower-energy configuration is always accepted at each step, while a higher-energy configuration is accepted only if the acceptance probability *P* = exp(−*dE/T*) is greater than a random number between 0 and 1, as illustrated in Figure 10 [52,53,54]. Even if the new configuration has higher energy (*dE*_j_ > 0), it is likely to be accepted in early iterations, owing to the high system temperature. As a cooling schedule decreases the system temperature in a controlled way (linearly or exponentially for example), the acceptance probability becomes lower and higher-energy states will seldom be accepted. In summary, SA initially searches a wide design space by allowing configurations that appear inferior at first glance, but it behaves like a steepest-gradient method in the end to narrow down to a local minimum. Through this transforming progress in SA, the initial ReCon configuration settles down to optimal configurations with minimum energy (fitness).

The allowable range of each variable and its initial value are listed in Table 10. The initial values were chosen in accordance with intuition obtained from the design of experiments (DOE) using LHS: The minimum height, the lowest altitude difference, smallest number of planes and satellites, and the greatest FoR. This initial configuration of x_0_ = [31/2, −200, 2, 1, 50] is used at the first step in Figure 10. The optimization process begins from the melted state, which crystallizes as temperature decreases exponentially by a factor of 10. Freezing happens when no or very few new configurations emerge for a consecutive number of iterations, less than five new configurations in three consecutive trials in this setting.

With this setup, the single-objective fitness function (*J*) is defined as a linear combination of figures of merit (*F*’s) and penalty terms (*h*’s) that are functions of the input design vector (***x***).

(17) $$J\left(x\right)=\sum_{i=1}^{4}w_{i}s_{i}F_{i}\left(x\right)+g\sum_{j=1}^{5}c_{j}h_{j}\left(x\right),$$

such that violating constraints will yield positive h values and increase the total fitness *F*. Even if the weighted sum of *F*’s may be small, large constraint violations will increase the total fitness which is deemed undesirable from optimization perspectives. Amongst four figures of merit in Table 11, only *F*_1_ has a negative sign in its definition because coverage should be maximized, and the other figures of merit should be minimized. Each figure of merit is multiplied by a corresponding scaling factor (*s_i_*) and a weight (*w_i_*). Scaling factors are used to prevent any figure of merit from dominating the others, and weights balance the relative importance of the four objectives. Similarly, each constraint term is multiplied by a relative scale factor (*c_j_*), and the sum of their linear combination is again multiplied by a global gain *g*, as summarized in Table 12.

**Figure 11:**
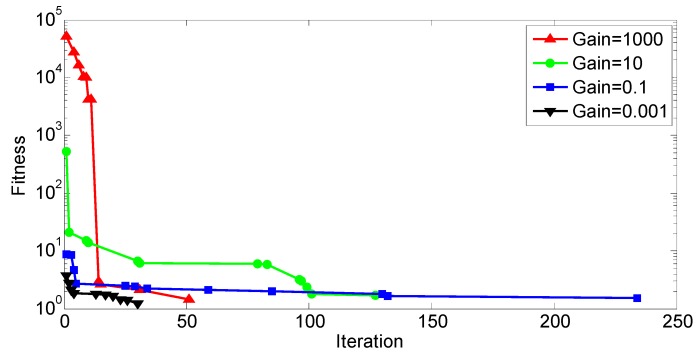
SA convergence history.

Figure 11 reports the time history of fitness where several SA runs were run with different penalty gains. As the gain grows, the optimizer tries to avoid constraint violation because any nonzero penalty will be amplified significantly. The lowest fitness was achieved with *g* = 0.001, but a penalty from constraint violation was non-zero. Therefore, *g* = 1000 was chosen for optimization which had the second lowest fitness and incurred no penalty. 

Table 13 and Table 14 summarize two types of optimal ReCon configuration obtained using SA. They differ in the number of orbital planes and the sign of *delta_alt*, but the magnitude of *delta_alt* and the field of regard are similar. The 3-plane solution has superior GOM coverage and ROM revisit time to the 5-plane solution, but its requirement of heavier constellation launch mass has resulted in a lower score (higher value of *J*). 

## 5. Genetic Algorithm

**Figure 12:**
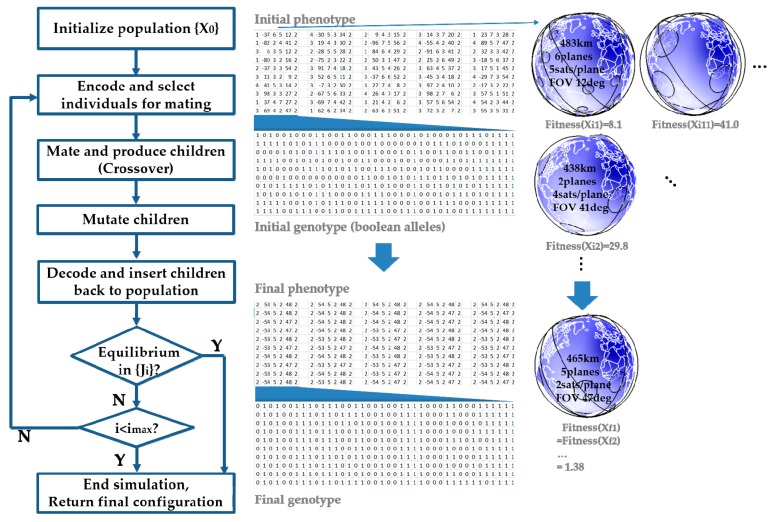
Genetic algorithm (GA) for ReCon optimization.

Evolutionary optimization mimics natural selection processes where individuals compete for survival in the population. Only the fittest can survive and reproduce, improving the entire population over generations. There are many algorithms falling into this category, depending on the forms which individuals may take: Genetic algorithm (gene sequences), genetic programming (solver programs), differential evolution (numeric vectors), neuroevolution (neural net weights), learning classifier system (rules or conditions) and so forth [55]. This study employs a genetic algorithm (GA) which is the most widely used amongst evolutionary optimization methods. Figure 12 explains the GA steps where randomized initial design variables of each individual are encoded into Boolean alleles for genetic operations (selection, crossover, mutation, and insertion) and decoded back for fitness evaluation prior to the next step or termination. Note that an initially diverse population reaches an equilibrium where the fittest (lowest fitness score) individuals with homogeneous characteristics constitute the final population.

In genotypes, each individual design is represented by a schema which is a template consisting of 0’s and 1’s [56]. The schema is a concatenation of binary forms of design variables, which are given a varying number of bits according to their values and required accuracy. Table 15 summarizes design variables and the number of bits to quantities them. Discrete variables don’t incur quantization errors in encoding or decoding, whereas continuous variables have quantization errors of (*maximum* − *minimum*)/2*^bits^* each time. Note also that the range of each variable has been modified from that used in SA such that GA can find an optimal solution faster. The genetic operation parameters are set up and tuned as follows. The selection process uses a roulette wheel selection scheme. A crossover rate of 0.95 and a mutation rate of 0.001 are used along with a population of 50. Experimentation has shown that higher mutation rates often lead to poor convergence because new, mutated species are continually injected into the population; on the other hand, lower mutation rates with a small population yield sub-optimal solutions as well because the whole population prematurely converges homogeneously to a sub-optimal solution.

**Figure 13:**
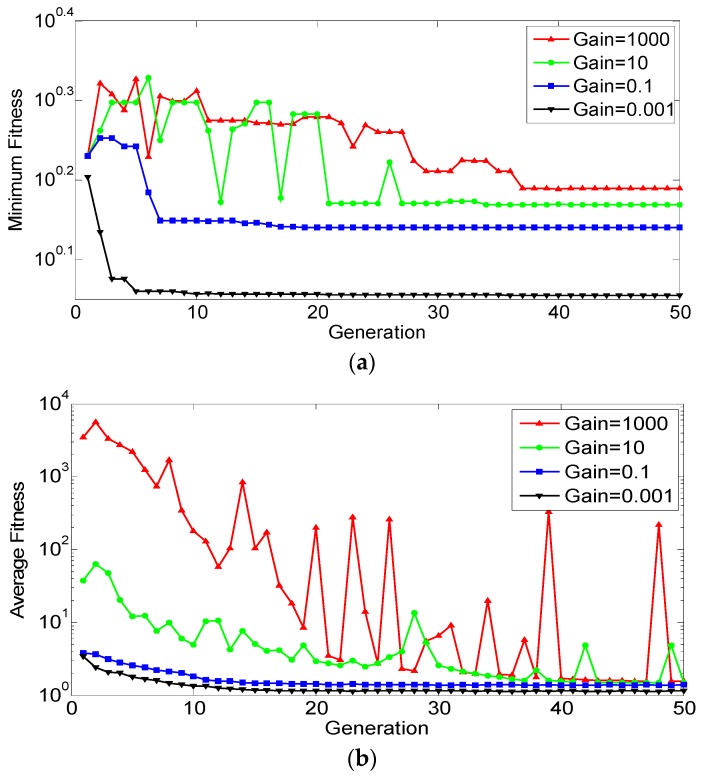
GA convergence history: (**a**) Population mean; (**b**) best individual.

Using the settings in Table 11 and Table 12, the same objective function as in SA (Equation 15) is to be minimized. Figure 13 shows the convergence history of the population mean fitness and the fitness of the best individual, respectively. If the constraint gain is too small (0.001), the constraint violation is discounted, and the resultant solution is sub-optimal. If the gain is too high (10 and 1000), the solution is also sub-optimal, so the most optimal solution is found to be 0.1.

**Figure A1:**
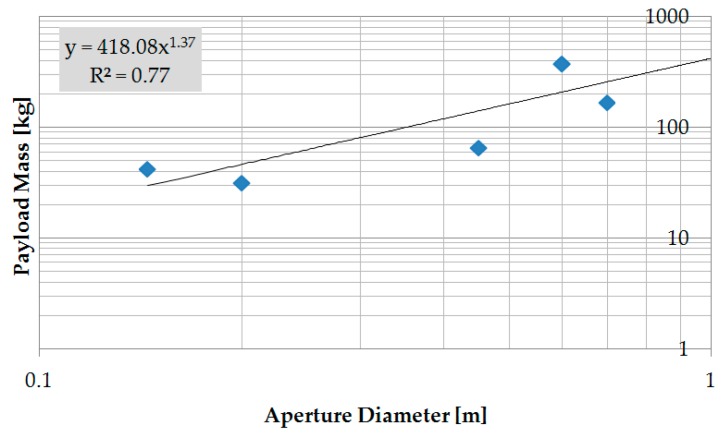
Sensor payload mass vs. aperture diameter.

Table 16 and Table 17 show similar GA trends, as already shown in SA, reaching either 3-plane or 5-plane optima in multiple runs. Overall GA achieves better score (lower *J*) by maintaining fine balances between performance and cost (mass). For example, the 3-plane GA solution has a negative *delta_alt*, resulting in a lower launch mass compared to the 3-plane SA solution with a positive *delta_alt*. The 5-plane GA solution has three satellites per plane, which also reduces the constellation launch mass compared to the 5-plane SA solution with four satellites per plane. In both cases, degradation in coverage or revisit performance is justified by savings in launch mass. The two GA solutions have nearly identical *J* score although the 5-plane solution is marginally better than the 3-plane solution. In either case, the individual satellite weighs 2 ton and the aperture has a diameter of 1.2 m, not significantly exceeding the ranges in the Earth-observation satellite database (Table A1 and Figure A1). The physical feasibility is thus verified, and the performance-to-cost (mass) analysis is conducted in the next section. 

## 6. Discussion

Both SA and GA produce very similar optimal solutions; both ReCon configurations have 5 orbit planes, 2 satellites per plane, and a field of regard of 47°. Only the altitude difference differs in sign, but the magnitudes are very close to each other. The quality of the GA solution is slightly better than the SA solution at the expense of greater computation time. A gradient-based optimization is also attempted and compared with SA and GA solutions via the time analysis. The sensitivity of the optimal solution and a case of Sun-synchronous orbits are also discussed.

### 6.1. Gradient-Based Optimization

**Figure 14:**
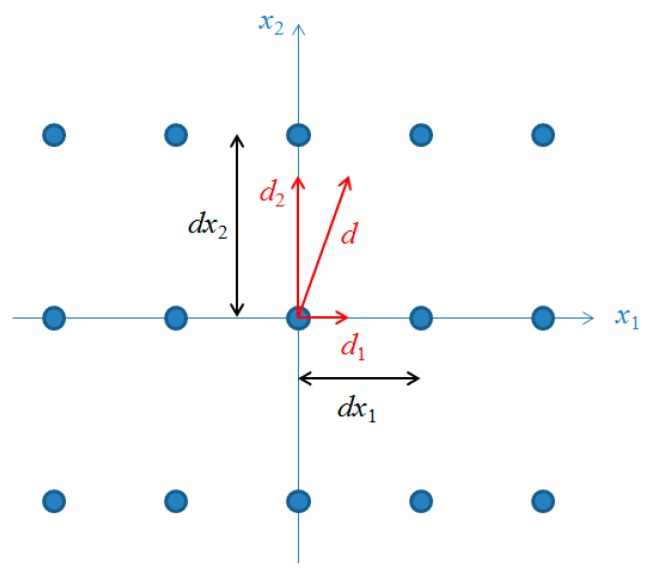
Illustration of modified step size calculation for discrete design variables.

In addition to the heuristic algorithm, ReCon optimization was also attempted using gradient-based methods. This was a significant challenge, given the fact that design space is a mixture of integer variables (n/k ratio, number of planes, number of satellites per plane) and continuous variables (Walker altitude difference, field of regard), making the problem inherently poorly suited to gradient-based methods. An approximation to the gradient was calculated via finite differences, as illustrated by vector ***d*** in Figure 14. There is generally no guarantee that the search direction will pass through grid points in the design space. Therefore, discrete variables were allowed to move in only one direction at a step, replacing ***d*** with ***d_2_*** in Figure 14, which has a larger projection than ***d_1_***. The algorithm can be considered a type of “pseudo-steepest descent” in which the general trend of the approximate gradient is followed in a step-wise manner to be compatible with the discrete design space. Because the line search step leads to “city block” style movements through the design space, methods like conjugate gradient would not help to provide a more direct trajectory. This simple version of steepest descent also makes a fair comparison against the heuristic algorithms (SA, GA) used in this study in their simplest form.

**Figure 15:**
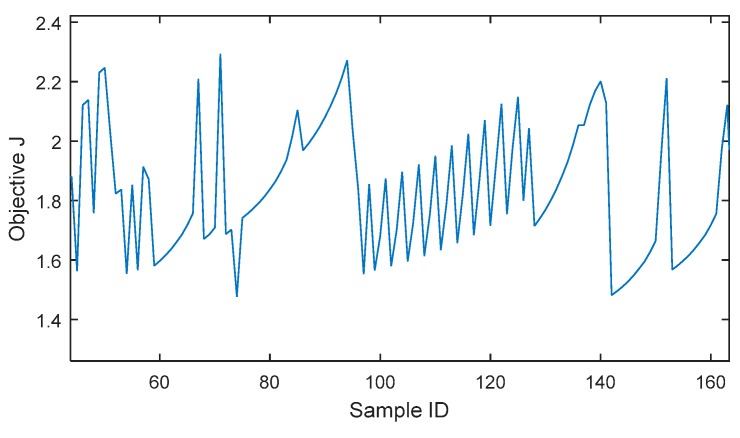
Illustration of objective (*J*) space for discrete samples.

Table 18 and Table 19 show the optimal solutions from the steepest descent (SD) method. The 2-plane SD solution has a score comparable to the 3-plane SA solution, and the 3-plane SD solution is a slightly better score than the 3-plane SA solution. However, these SD solutions could be obtained by starting from the optimal results of SA. Because the ReCon problem is extremely poorly conditioned for gradient-based methods, even the solution convergence itself was rarely achieved when LHS results or random initialization were used as starting points. Figure 15 illustrates a snapshot of objective space for coarsely sampled discrete points similar to Figure 14. Even in converged cases, the scores of SD solutions were still worse (higher *J*) than GA solutions. 

**Figure 16:**
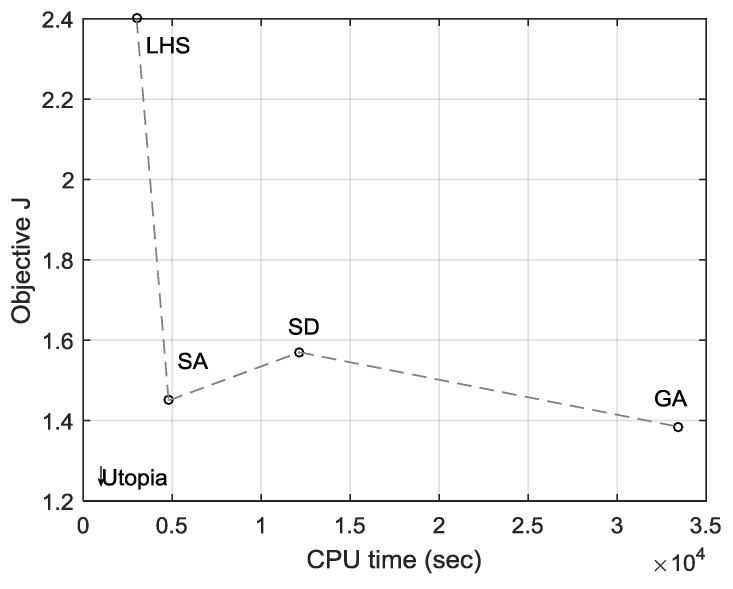
Time taken by each method and solution optimality.

Figure 16 is a representation of time versus quality analysis for solutions obtained by LHS (Section 3), SD (Section 6.1), SA (Section 4), and GA (Section 5) methods [57]. A desktop with Intel^®^ Core™ i7-2600 CPU (3.40 GHz) and 16.0 GB RAM was used in the experiments. LHS orthogonally sampled 100 designs, resulting in the lowest quality (highest *J*) among the four but taking the shortest time. SA shows a considerable improvement in solution quality compared to the LHS solution while taking less than twice the time required by LHS. SD took a longer time than SA and yielded an inferior solution because gradient-based methods are ill-suited to the ReCon problem. The GA solution is slightly (4%) more optimal than the SA solution in terms of the *J* value. However, GA required 7 times longer computation time than SA because GA performs optimization over a population whereas SA optimizes a single design point. With SD solution dominated by the rest, the other three methods constitute a non-dominated Pareto front whose time-quality tradeoff may be utilized throughout a design project from its early prototyping to final refinement stages. Future work could employ other population-based methods (e.g. ant colony optimization) as candidates to achieve similar performance as GA while reducing computation time.

Direct comparison with static satellite constellations is not straightforward because they cannot adopt the “choice and concentration” observation strategy of a ReCon; a static constellation cannot lock its satellite orbits after a ground event occurs, and the orbits will keep on approaching and drifting away from the target of interest. Continuing our discussion anyway, LandSat has a revisit time of one to two weeks, so a reconfiguration (lock-on) time of 3 days would require doubling the number of ReCon’s orbit planes in Table 16 for a static constellation. The number of satellites, and hence the total constellation mass is doubled. Using the objective computation formula, a static constellation would have a *J* score of 2 at least. It is thus shown that the performance-to-cost (mass) analysis favors a ReCon over a satellite constellation.

### 6.2. Sensitivity Analysis

A sensitivity analysis is conducted at the optimal design from GA, x* = (15/1, −54.7, 5, 2, 47.1), where the local gradient is obtained at the optimal point followed by normalization. As shown in Equation (18), the local gradient vector of fitness, ∇*J*, is obtained by incrementing one design variable at a time and dividing fitness difference by that increment. The local gradient vector is normalized through entrywise multiplication (∘) with a scaled vector, x* divided by fitness at x* [9].

(18) $$\begin{array}{c} \nabla \bar{J}=\frac{x^{*}}{J\left(x^{*}\right)}\circ \nabla J=\frac{1}{J\left(x^{*}\right)}{\left[\begin{array}{c} N\\ A\\ P\\ S\\ R \end{array}\right]}^{*}\circ {\left[\begin{array}{c} \partial J/\partial N\\ \partial J/\partial A\\ \partial J/\partial P\\ \partial J/\partial S\\ \partial J/\partial R \end{array}\right]}^{*}=\frac{1}{J\left(x^{*}\right)}{\left[\begin{array}{c} N\partial J/\partial N\\ A\partial J/\partial A\\ P\partial J/\partial P\\ S\partial J/\partial S\\ R\partial J/\partial R \end{array}\right]}^{*}\\ \mathrm{where}\;{\left[\begin{array}{c} \partial J/\partial N\\ \partial J/\partial A\\ \partial J/\partial P\\ \partial J/\partial S\\ \partial J/\partial R \end{array}\right]}^{*}≅[\begin{array}{c} (J(N^{*}+\Delta N,A^{*},P,S^{*},R^{*})-J(N^{*},A^{*},P^{*},S^{*},R^{*}))/\Delta N\\ (J(N^{*},A^{*}+\Delta A,P^{*},S^{*},R^{*})-J(N^{*},A^{*},P^{*},S^{*},R^{*}))/\Delta A\\ (J(N^{*},A^{*},P+\Delta P,S^{*},R^{*})-J(N^{*},A^{*},P^{*},S^{*},R^{*}))/\Delta P\\ (J(N^{*},A^{*},P^{*},S^{*}+\Delta S,R^{*})-J(N^{*},A^{*},P^{*},S^{*},R^{*}))/\Delta S\\ (J(N^{*},A^{*},P,S^{*},R^{*}+\Delta R)-J(N^{*},A^{*},P^{*},S^{*},R^{*}))/\Delta R \end{array}] \end{array}$$

**Figure 17:**
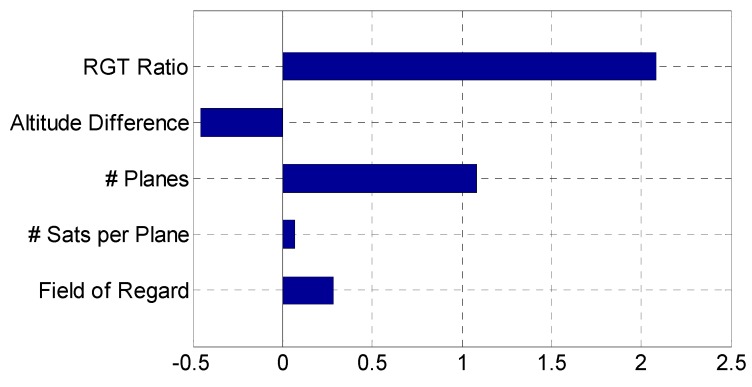
Normalized sensitivity of design variables (RGT ratio divided by 10).

Table 20 and Figure 17 show that the RGT ratio has the largest impact (highest sensitivity), followed by the number of planes. Because both variables have positive values, increasing these variables will reduce the optimality of a ReCon. In fact, increasing the RGT ratio by 0.5 decreases the RGT altitude by 100 km approximately, which increases fuel consumption to compensate for atmospheric drag and to keep satellites in place. Due to a similar reason, it makes intuitive sense that the number of satellites in the constellation would drive the objective function. The sensitivity of the altitude difference was negative at this design point, which means that increasing this variable will improve the optimality.

**Figure 18:**
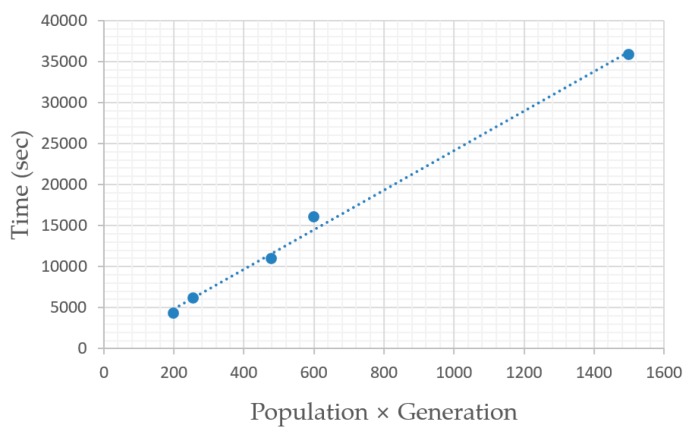
GA problem size and time taken for optimization.

Figure 18 shows a relationship between the GA problem size and computation time. The problem size equals the number of generations times the number of individuals within a population. It can be inferred that computation time linearly increases with the problem size. As for parameter setting, the mutation rate of 0.001 was increased to 0.002 and 0.004, leading to increased average *J* scores of 1.73 and 2.30 with a statistically significant *p*-value at 0.0 (significance level 0.05).

### 6.3. Sun-Synchronous Orbits

Now that SA and GA have been shown to produce very similar results, either method may be used per usage: SA for fast-prototyping and GA for fine-tuning, for example. Most traditional Earth observation satellites have been using Sun-synchrnous orbits (SSOs), although small-satellite swarms released from International Space Station have non-SSOs. Advances in image processing technologies also help the correction of nonhomogeneous solar illumination of satellite imagery provided by this type of swarms. One embodiment of the ReCon framework employs SSOs, and the SA results are summarized in Table 21. The number of planes is the same, but the satellite per plane has increased from 2 to 3 because a wider latitude band needs to be covered with near-polar SSOs. This has also resulted in higher altitudes than non-SSOs, as can been seen from the RGT altitude of 720 km in ROM and at the Walker altitude of 740 km (= 720 − ( − 20)) in GOM. The optimizer achieved high GOM area coverage and short ROM revisit intervals at the expense of heavy constellation mass and long reconfiguration time. Weights of performance metrics may be adjusted to improve this ReCon design in terms of mass and reconfiguration time.

## 7. Conclusions

This paper proposes a reconfigurable satellite constellation (ReCon) for Earth observation. Its physical feasibility is demonstrated using the payload aperture and chemical propellant database from past missions. On top of that, a ReCon is shown to provide better performance-to-mass ratios than static constellations for uncertain ground targets. Its high responsiveness can be useful in particular, for example, to observe extreme weather events now occurring more often and unexpectedly across the globe. Its complexity in constellation management is currently overcome by active research in academia and industry [19,20,21].

Systems engineering approaches with heuristic optimization methods are exploited, whose procedures and results are discussed in detail. The optimization goal is to balance coverage, response time, and constellation mass which is a proxy of cost. Genetic algorithm yields more optimal solutions than simulated annealing and steepest gradient methods, due to the nonlinearity of the problem.

To realize a ReCon which is a complex federated satellite system, political and policy aspects must also be considered [58,59,60]. Multi-sensor satellite constellations may be integrated by multinational agencies (A-Train) or operated by several entities (Disaster Monitoring Constellation) [61]. Future work will address these issues in addition to the technical expansion of the current ReCon framework. Areas of further technical research includes, but not limited to, investigation of low-thrust (electric propulsion), radar observation and GPS radio occultation, and satellite constellations for planetary observation [62,63]. 

## Figures and Tables

**Table 1:** Figures of merit and constraints in ReCon optimization.

Figure of Merit	Definition	Constraint	Definition
*F*_1_ *F*_2_ *F*_3_ *F*_4_	(−1) × GOM coverage (%) ROM revisit time (s) Constellation mass (kg) Reconfiguration time (day)	*h*_1_ *h*_2_ *h*_3_ *h*_4_	Minimum altitude (km) Maximum altitude (km) Maximum aperture (m) Max propellant mass fraction

**Table 2:** Design variables in ReCon simulation.

Design Variable	Description	Range	Unit
*nk*	Repeat ground track (RGT) ratio (τ)	{31/2, 15/1, 29/2, 14/1}	-
*delta_alt* *n_planes* *n_sats* *regard* *prop*	Walker altitude difference from RGT altitude # of planes in Walker constellation # of satellites per orbit plane Field of regard Propulsion type	[−50, 50] {2, 3, 4, 5, 6, 7, 8, 9} {1, 2, 3, 4, 5} [5, 50] {cold gas, monoprop, biprop} ^1^	km - - ° -

^1^ monopropellant and bpropellant.

**Table 3:** Internal variables in ReCon simulation.

Internal Variable	Description	Unit
*aperture*	Optical telescope aperture diameter	m
*fl* *prop_dry_mas* *s* *propellant_mass* *optics_mass* *rgt_alt* *delta_v* *sat_dry_mass*	Optical telescope focal length Propulsion system dry mass Propellant mass Optical subsystem mass Repeating groundtrack altitude Total lifetime fuel burn Satellite dry mass	m kg kg m km m/s kg

**Table 4:** Parameters used in ReCon simulation.

Parameter	Description	Value	Unit
*life*	Orbit lifetime	5	Year
*e* *walker_phase* *inc* *n_recons* *gsd* *regional_lat* *global_lat_band*	Orbit eccentricity Walker phasing parameter Orbit inclination # of reconfigurations over lifetime Ground sample distance Regional latitude of interest Global latitude band of interest	0 1 60 10 0.5 55 [0, 60]	- - ° - m ° °

**Table 5:** Constraints imposed in ReCon simulation.

Constraint	Description	Value	Unit
*min_alt*	Minimum altitude	350	km
*max_alt* *max_regard* *max_aperture* *max_prop_frac*	Maximum altitude Maximum field of regard Maximum aperture diameter Maximum propellant mass fraction	1200 50 1.8 0.3	km ° m -

**Table 6:** Objectives of ReCon simulation.

Objective	Description	Unit
*rom_revisit*	ROM revisit time	s
*gom_coverage* *reconfg_time* *const_mass*	GOM temporal coverage Reconfiguration time between GOM and ROM Constellation total mass	% days kg

**Table 7:** Inputs and output variables of simulation modules.

Module	Input Variables	Output Variables
Astrodynamics	*nk, delta_alt, n_planes, n_sats, regard,* *e, walker_phase, inc*	*rgt_alt, rom_revisit, gom_revisit*
Optics Maneuvers Propulsion	*regard, delta_alt, fov, gsd, rgt_alt* *prop, delta_alt, life, n_recons, rgt_alt, area* *prop, sat_dry_mass, delta_v*	*optics_mass, aperture* *delta_v, reconfig_time* *prop_dry_mass, propellant_mass*
Constellation	*n_planes, n_sats, optics_mass, aperture,* *prop_dry_mass, propellant_mass*	*sat_dry_mass, const_mass, area*

**Table 8:** Factors and Levels used in Latin Hypercube Sampling.

Design Variable	Description	Factor	#Levels	Units
*nk*	RGT ratio	N	4	-
*delta_alt* *n_planes* *n_sats* *regard*	Altitude difference between Walker and RGT Number of orbit planes Number of satellites per plane Field of regard	A P S R	8 4 4 8	km - - °

**Table 9:** Main effects from Latin Hypercube Sampling with 100 design points.

Factor/ Level	Value of Level [Unit]	△ROM Revisit [sec]	△GOM Revisit [sec]	△Constellation Mass [kg]	△Reconfig Time [day]
N1 N2 N3 N4	31/2 15/1 29/2 14/1	−667 −1658 +2739 +317	−7800 −479 +11,797 −1969	−2444 −246 +1005 +2962	−0.1 −3.4 +0.8 +2.1
A1 A2 A3 A4 A5 A6 A7 A8	−40 km −30 km −20 km −10 km 10 km 20 km 30 km 40 km	−3522 −3625 −858 +561 +1021 +565 +1080 +8243	−22,931 −25,629 −332 +6831 +5863 +6457 +8794 +37,467	+6775 +2482 −262 −424 −1462 −1953 −323 −4216	−6.7 −5.2 −0.4 +10.9 +8.6 −1.5 −5.5 −7.0
P1 P2 P3 P4	1 plane 2 planes 3 planes 4 planes	+428 −489 +1098 −598	−5662 −7380 +7839 +6207	−912 +479 +217 +162	−0.2 −0.4 −1.4 +1.4
S1 S2 S3 S4	2 sats 3 sats 4 sats 5 sats	+3956 −924 −1423 +1584	+19,599 −3003 −6482 −10,062	−4722 +448 +1052 +2239	3.2 −2.7 −1.6 +0.4
R1 R2 R3 R4 R5 R6 R7 R8	5° 10° 15° 20° 25° 30° 35° 40°	+5072 +1798 −734 +1205 −1122 −1971 −1648 −2995	+44,821 +7602 +368 +9709 −14,134 −17,839 −16,690 −20,366	+491 +2281 +738 −2154 +1518 −681 −972 498	+2.7 +6.2 +0.5 −2.4 −4.0 −2.3 +0.9 −1.1

**Table 10:** Range and initial value of design variables in SA.

Design Variable	Description	Range	Initial Value	Type
*nk*	RGT ratio	[13/1, 31/2]	31/2	Discrete
*delta_alt* *n_planes* *n_sats* *regard* *prop*	Altitude difference Number of orbital planes Number of satellites per plane Field of regard (FoR) Propellant Type	[−200, 200] [2, 9] [1, 5] [5, 50] Monopropellant	−200 km 2 1 50° -	Continuous Integer Integer Continuous -

**Table 11:** Figures of merit, scaling and weighting factors in single-objective optimization.

Figure of Merit	Definition	Typical Value	Scaling (*s_i_*)	Weighting (*w_i_*)
*F*_1_ *F*_2_ *F*_3_ *F*_4_	(−1) × GOM coverage (%) ROM revisit time (s) Constellation mass (kg) Reconfiguration time (day)	−5 1000 10,000 2	0.5 0.001 0.0001 1	0.25 0.25 0.30 0.20

**Table 12:** Constraints, scaling factors, and gains in single-objective optimization.

Constraint	Definition	Typical Value	Scaling (*s_i_*)	Gain (*w_i_*)
*h*_1_ *h*_2_ *h*_3_ *h*_4_	Minimum altitude (km) Maximum altitude (km) Maximum aperture (m) Max propellant mass fraction	350 1200 1.8 0.3	0.5 0.001 0.0001 1	0.1

**Table 13:** SA optimal solution (3 planes).

Type	Symbol	Description	Optimum
Design variable	*nk* *delta_alt* *n_planes* *n_sats* *regard*	RGT ratio Altitude difference Number of orbital planes Number of satellites per plane Field of regard	15/1 −42.9 km 3 5 47.8°
Performance metrics	*F*_1_ *F*_2_ *F*_3_ *F*_4_ *J*	GOM area coverage ROM revisit time Constellation mass Reconfiguration time Objective function	3.32 % 1018 sec 32,796 kg 3.13 day 1.570

**Table 14:** SA optimal solution (5 planes).

Type	Symbol	Description	Optimum
Design variable	*nk* *delta_alt* *n_planes* *n_sats* *regard*	RGT ratio Altitude difference Number of orbital planes Number of satellites per plane Field of regard	15/1 49.6 km 5 2 46.8°
Performance metrics	*F*_1_ *F*_2_ *F*_3_ *F*_4_ *J*	GOM area coverage ROM revisit time Constellation mass Reconfiguration time Objective function	2.89 % 1609 sec 26,276 kg 3.17 days 1.463

**Table 15:** Range and initial value of design variables in GA.

Design Variable	Range	Bits	Type
RGT ratio	[13/2, 14/1]	4	Discrete
Altitude difference Number of orbital planes Number of satellites per plane Field of regard (FoR) Propellant Type	[−100, 100] [2, 7] [1, 7] [5, 60] -	12 4 4 12 -	Continuous Integer Integer Continuous Fixed

**Table 16:** GA optimal solution (3 planes).

Type	Symbol	Description	Optimum
Design variable	*nk* *delta_alt* *n_planes* *n_sats* *regard*	RGT ratio Altitude difference Number of orbital planes Number of satellites per plane Field of regard	15/1 −53.9 km 3 4 46.8°
Performance metrics	*F*_1_ *F*_2_ *F*_3_ *F*_4_ *J*	GOM area coverage ROM revisit time Constellation mass Reconfiguration time Objective function	1.95 % 1346 sec 25,187 kg 2.92 days 1.385

**Table 17:** GA optimal solution (5 planes).

Type	Symbol	Description	Optimum
Design variable	*nk* *delta_alt* *n_planes* *n_sats* *regard*	RGT ratio Altitude difference Number of orbital planes Number of satellites per plane Field of regard	15/1 −54.7 km 5 2 47.1°
Performance metrics	*F*_1_ *F*_2_ *F*_3_ *F*_4_ *J*	GOM area coverage ROM revisit time Constellation mass Reconfiguration time Objective function	1.95 % 1602 sec 21,318 kg 2.93 days 1.382

**Table 18:** SD optimal solution (3 planes).

Type	Symbol	Description	Optimum
Design variable	*nk* *delta_alt* *n_planes* *n_sats* *regard*	RGT ratio Altitude difference Number of orbital planes Number of satellites per plane Field of regard	15/1 −100 km 3 4 30°
Performance	*J*	Objective function	1.565

**Table 19:** SD optimal solution (2 planes).

Type	Symbol	Description	Optimum
Design variable	*nk* *delta_alt* *n_planes* *n_sats* *regard*	RGT ratio Altitude difference Number of orbital planes Number of satellites per plane Field of regard	15/1 −90 km 2 5 37°
Performance	*J*	Objective function	1.570

**Table 20:** Sensitivity at the optimal design point.

Design Variable *x*	Step Size △*x*	Optimal Fitness J(x*)	Perturbed Fitness J(x* + △*x*)	Partial Derivative ∂*J*/∂*x*	Normalized Sensitivity ▽*J*(x*)x*/*J*(x*)
RGT ratio	0.5	1.382	2.343	1.922	20.87
Altitude difference Number of orbital planes Number of satellites per plane Field of regard (FoR)	10 km 1 1 5°		1.498 1.681 1.428 1.423	0.012 0.299 0.046 0.008	−0.458 1.083 0.067 0.284

**Table 21:** Sun-synchronous orbits (SSO) ReCon optimization (SA).

	Symbol	Description	
Design variable	*nk* *delta_alt* *n_planes* *n_sats* *regard*	RGT ratio Altitude difference Number of orbital planes Number of satellites per plane Field of regard	29/2 −19.9 km 5 3 41.4°
Performance metrics	*J*_1_ *J*_2_ *J*_3_ *J*_4_	GOM area coverage ROM revisit time Constellation mass Reconfiguration time	4.71 % 1173 sec 41796 kg 13.6 days

**Figure A2:**
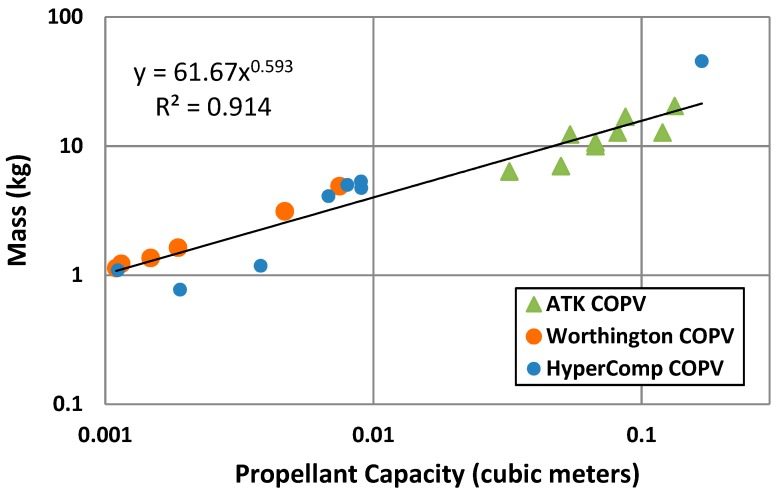
Tank mass vs. tank volume.

## Appendix A. Repeat Ground Tracks

A satellite’s angular speed and its orbit orientation is a function of time and orbit parameters describing the orbit shape and size. The time derivatives of the satellite speed and the orbit orientation are used to define the repeat ground track (RGT) ratio, as shown in Equation (A1).

(A1) $$\tau =\frac{N_{S}}{N_{D}}=\frac{T_{G}}{T_{S}}=\frac{2\pi /\left(\omega_{E}-\dot{\Omega }\right)}{2\pi /\left(\dot{M}+\dot{\omega }\right)}=\frac{\dot{M}+\dot{\omega }}{\omega_{E}-\dot{\Omega }}=\frac{n+\Delta n+\dot{\omega }}{\omega_{E}-\dot{\Omega }}$$

where *N_D_* is the repeating cycle length in days, Ns is the number of satellite revolutions made the during that repeating cycle, *T_G_* is the nodal period of Greenwich, *T*_s_ is the orbital period of a satellite. The orbital rates are defined as follows: *n* is the mean motion of a satellite, *dM/dt* ( = *n + Δn*) is the perturbed mean motion, *dω/dt* is the drift rate of the argument of perigee, due to perturbations, *ω*_E_ is the rotation rate of the Earth, and *dΩ/dt* is the nodal regression rate. Based on the definition of *n* = (*μ/a*)^1/2^, these orbital rates are represented as the sum of a periodic term and a secular term each:

(A2) $$\frac{\dot{\Omega }}{n}=-\frac{3J_{2}R_{E}^{2}}{2p^{2}}c-\frac{9J_{2}^{2}}{4p^{4}}c\left[\left(\frac{3}{2}+\frac{1}{6}e^{2}+e^{\prime }\right)-\left(\frac{5}{3}-\frac{5}{25}e^{2}+\frac{3}{2}e^{\prime }\right)+O\right],$$

(A3) $$\frac{\dot{\omega }}{n}=\frac{3J_{2}R_{E}^{2}}{2p^{2}}\left(\frac{5}{2}s^{2}-2\right)+\frac{9J_{2}^{2}R_{E}^{4}}{p^{2}}\left[\left(4+\frac{7}{12}e^{2}+2e^{\prime }\right)-s^{2}\left(\frac{103}{12}+\frac{3}{8}e^{2}+\frac{11}{2}e^{\prime }\right)+O\right]$$

(A4) $$\frac{\Delta n}{n}=\frac{3J_{2}R_{E}^{2}}{2p^{2}}\left(1-\frac{3}{2}s^{2}\right)e^{\prime }+\frac{9J_{2}^{2}R_{E}^{4}}{4p^{2}}\left[\frac{1}{2}{\left(1-\frac{3}{2}s^{2}\right)}^{2}e^{\prime }+\left(\frac{5}{2}+\frac{10}{3}e^{2}\right)-s^{2}\left(\frac{19}{3}+\frac{26}{3}e^{2}\right)+O\right],$$

where *s* = *sin,* *s* = *sin i*, *p*, and *e*’ = (1−*e*^2^)^1/2^. The symbol *O* represents higher-order terms depending on *e^4^* and *J_4_/J_2_^2^* which may be omitted for near-circular, Earth-centric orbits. Also, the second terms in the above equations negligible compared to the first terms for short-duration missions.

## Appendix B. Satellite Aperture and Tank Mass Data

This appendix provides charted data of sensor payload mass and propellant tank mass as functions of aperture diameter. First, Figure A1 plots the relationship between aperture diameter and payload mass (Equation (8)). The aperture diameter and mass are summarized in Table A1.

**Table A1:** Figures of merit, scaling and weighting factors in single-objective optimization.

Mission	Payload	Vendor	Aperture (m)	Payload Mass (kg)
RapidEye TopSat OrbView-3 Quickbird WorldView-1 Ikonos GeoEye-1	REIS RALCam 1 OHRIS BHRC 60 WV 60 OSA GIS	Jena-Optronik MDA Northrop Grumman ITT Exelis ITT Exelis Kodak ITT Exelis	0.145 0.2 0.45 0.6 0.6 0.7 1.1	43 32 66 380 380 171 452

Figure A2 present the relationship between tank mass and tank volume for cold gas (Equation (14)). A very similar relation is obtained for monopropellant as well (Equation (15)).

## Appendix C. Fuel (“Delta-V”) Budget

This appendix explains how delta-v, a measure of fuel consumption from satellite maneuvers, is budgeted in a ReCon framework. First, Equation (A1) calculates delta-v for the deployment stage from a parking orbit to a GOM orbit.

(A5) $$\begin{array}{r} \Delta V_{c}=\left|\sqrt{\frac{\mu_{E}}{R_{E}+h_{RGT}+\Delta h}}-\sqrt{\frac{2\mu_{E}}{R_{E}+h_{RGT}+\Delta h}-\frac{2\mu_{E}}{\left(R_{E}+h_{RGT}+\Delta h\right)+\left(R_{E}+h_{P}\right)}}\right|\\ +|\sqrt{\frac{\mu_{E}}{R_{E}+h_{P}}}-\sqrt{\frac{2\mu_{E}}{R_{E}+h_{RGT}}-\frac{2\mu_{E}}{\left(R_{E}+h_{RGT}+\Delta h\right)+\left(R_{E}+h_{P}\right)}}|, \end{array}$$

The delta-v for reconfiguration is obtained by multiplying the delta-v per maneuver and the number of reconfiguration maneuvers between GOM and ROM:

(A6) $$\begin{array}{c} \Delta V_{r}=N_{r}\left|\sqrt{\frac{\mu_{E}}{R_{E}+h_{RGT}+\Delta h}}-\sqrt{\frac{2\mu_{E}}{R_{E}+h_{RGT}+\Delta h}-\frac{2\mu_{E}}{2\left(R_{E}+h_{RGT}\right)+\Delta h}}\right|+|\sqrt{\frac{\mu_{E}}{R_{E}+h_{RGT}}}-\\ \sqrt{\frac{2\mu_{E}}{R_{E}+h_{RGT}}-\frac{2\mu_{E}}{2(R_{E}+h_{RGT})+\Delta h}}|, \end{array}$$

Stationkeeping delta-v consists of an atmospheric drag term and a solar radiation pressure term, both of which are proportional to lifetime.

(A7) $$\Delta V_{s}=\Delta V_{atm}+\Delta V_{solar}=N_{year}\left(\frac{\pi C_{D}A}{m}\rho_{atm}av\frac{365\times 24\times 60\times 60}{T}+30\right),$$

Lastly, Equation (A4) defines the delta-v for decommissioning at the end of a satellite’s life.

(A8) $$\Delta V_{d}=\left|\sqrt{\frac{\mu_{E}}{R_{E}+h_{RGT}+\Delta h}}-\sqrt{\frac{2\mu_{E}}{R_{E}+h_{RGT}+\Delta h}-\frac{2\mu_{E}}{\left(R_{E}+h_{RGT}+\Delta h\right)+\left(R_{E}+h_{D}\right)}}\right|$$
